# Current developments and future perspectives of nanotechnology in orthopedic implants: an updated review

**DOI:** 10.3389/fbioe.2024.1342340

**Published:** 2024-03-18

**Authors:** Wenqing Liang, Chao Zhou, Juqin Bai, Hongwei Zhang, Hengguo Long, Bo Jiang, Haidong Dai, Jiangwei Wang, Hengjian Zhang, Jiayi Zhao

**Affiliations:** ^1^ Department of Orthopaedics, Zhoushan Hospital of Traditional Chinese Medicine Affiliated to Zhejiang Chinese Medical University, Zhoushan, China; ^2^ Department of Orthopedics, Zhoushan Guanghua Hospital, Zhoushan, China; ^3^ Rehabilitation Department, Zhoushan Hospital of Traditional Chinese Medicine Affiliated to Zhejiang Chinese Medical University, Zhoushan, China; ^4^ Medical Research Center, Zhoushan Hospital of Traditional Chinese Medicine Affiliated to Zhejiang Chinese Medical University, Zhoushan, China

**Keywords:** nanomaterials, osseointegration, biocompatibility, bone fractures and defects, orthopaedic implants

## Abstract

Orthopedic implants are the most commonly used fracture fixation devices for facilitating the growth and development of incipient bone and treating bone diseases and defects. However, most orthopedic implants suffer from various drawbacks and complications, including bacterial adhesion, poor cell proliferation, and limited resistance to corrosion. One of the major drawbacks of currently available orthopedic implants is their inadequate osseointegration at the tissue-implant interface. This leads to loosening as a result of immunological rejection, wear debris formation, low mechanical fixation, and implant-related infections. Nanotechnology holds the promise to offer a wide range of innovative technologies for use in translational orthopedic research. Nanomaterials have great potential for use in orthopedic applications due to their exceptional tribological qualities, high resistance to wear and tear, ability to maintain drug release, capacity for osseointegration, and capability to regenerate tissue. Furthermore, nanostructured materials possess the ability to mimic the features and hierarchical structure of native bones. They facilitate cell proliferation, decrease the rate of infection, and prevent biofilm formation, among other diverse functions. The emergence of nanostructured polymers, metals, ceramics, and carbon materials has enabled novel approaches in orthopaedic research. This review provides a concise overview of nanotechnology-based biomaterials utilized in orthopedics, encompassing metallic and nonmetallic nanomaterials. A further overview is provided regarding the biomedical applications of nanotechnology-based biomaterials, including their application in orthopedics for drug delivery systems and bone tissue engineering to facilitate scaffold preparation, surface modification of implantable materials to improve their osteointegration properties, and treatment of musculoskeletal infections. Hence, this review article offers a contemporary overview of the current applications of nanotechnology in orthopedic implants and bone tissue engineering, as well as its prospective future applications.

## 1 Introduction

Nanotechnology is a broad field wherein chemical, physical, and biological properties, as well as the structures of materials, can be changed and shaped at the nano-level. Nanomaterials display size-dependent attributes which are rarely observed in bulk matter. Top-down and bottom-up methodologies are commonly employed for nanoparticle synthesis. The top-down methodology encompasses physical involvement strategies, including thermal evaporation pyrolysis, physical vapor deposition (PVD), mechanical machining, and lithography (Baptista et al., 2018). Bottom-up methods consist of chemical and biological approaches. Sol-gel, chemical co-precipitation, hydrothermal method, chemical vapor deposition (CVD), micro-emulsions, sonochemical, and microwave methods are involved in the bottom-up chemical approaches (Shah, 2014). Additionally, plant extracts, enzymes, agricultural waste, microorganisms, and actinomycetes are all viable possibilities for the synthesis of nanoparticles (Niemeyer and Mirkin, 2004). Recent developments in nanotechnology have enabled a wide range of novel applications in various fields, including environmental science (Kumar V. et al., 2019; Kumar et al., 2019b), biotechnology (Kumar et al., 2017a; Rani et al., 2018), agriculture and food industry (Mobasser and Firoozi, 2016), molecular biology (Ulijn and Jerala, 2018; Kumar et al., 2019c), and medicine (Guerra et al., 2018; Kailasa et al., 2021; Wang S. et al., 2022; Shahcheraghi et al., 2022). The field of nanomedicine, which is an application of nanotechnology to medicine, has resulted in the development of several procedures to diagnose, prevent, and manage a vast variety of diseases, such as imaging in medicine, the development of scaffolds for tissue engineering, drug distribution, immunotherapy, and tumor therapy. Due to their molecular origin, biological systems and pathophysiologic processes are fundamentally constituted of nanoscale components. The tremendous potential of employing nanotechnology in medicine originates from this fundamental fact (Silva, 2004). This hybrid discipline has been designated with various terminologies, including bio-nanotechnology, biomedical nanotechnology, and nanomedicine. The leading application of nanotechnology in medicine includes drug delivery in the pharmaceutical industry: for example, nanoscale polymer capsules designed for controlled breakdown and release of drugs (Emerich and Thanos, 2003), and target-specific diagnostic nanoparticle pharmaceuticals for use in medical imaging (Shen et al., 1993). Similarly, nanotechnology has enabled several novel orthopedic treatments Nanomaterials have emerged as promising candidates for the fabrication of orthopedic implants (Wang et al., 2016; Cheng et al., 2018a) due to their ability to imitate or mimic the structure of bone. Bone substitutes are essential in orthopedic applications to treat irreversible damage to healthy, natural bone. Nanomaterials are anticipated to play a pivotal role in this scenario by modulating cell migration, differentiation, and proliferation in addition to providing structural support to cells (referred to as nanofunctionalized scaffolding (Ferraris et al., 2016; Antoniac et al., 2022; Feltz et al., 2022).

Bone is an active tissue that undergoes constant transformation, and in the instance of a fracture, bone cannot re-acquire and regenerate its pre-injury mechanical and physiological properties (Duda et al., 2023). The skeletal system is resilient (i.e., capable of enduring shock without experiencing permanent deformation or rupture), but it can be damaged by certain accidents, disorders, and diseases. The severity of the mortality varies for various bones, but all of the resulting defects and injuries in the skeletal structure can contribute to increased mortality (Adam et al., 2020; Gao et al., 2023). Bioimplants have surfaced as a potentially useful treatment solution for a wide variety of conditions, including cardiovascular disease, visual impairments, neurological disorders, dental disorders, disfigurement, and orthopedic issues (Ghezzi et al., 2013; Song et al., 2013; Jackson, 2016; Scaini and Ballerini, 2018). A great deal of literature describes engineering techniques that mimic the physical and chemical properties and gradient architecture of real organs and tissues using common metallic and non-metallic materials. Nonetheless, there are certain limitations associated with conventional bioimplants. They are incompatible with tissues and react infrequently with them; the human body does not always tolerate them (Bian et al., 2016). The implant sector has been greatly influenced by nanotechnology in the past few years. Researchers are motivated to investigate the potential of nanomaterials with biologically inspired features to enhance the effectiveness of traditional implants. When designing such implants, it is necessary to consider the biocompatibility of the material along with its chemical characteristics, surface properties, mechanical properties, and failure properties. This is done to ensure that the implant mimics the physiological characteristics of bone and merges with the surrounding tissue meanwhile retaining its integrity in the process.

## 2 Nanotechnology-based orthopaedic implants

The potential of nanotechnology to revolutionize the field of orthopaedics depends on its ability to produce joint replacements and implants with increased durability. Nanomaterials with modified physicochemical characteristics (i.e., smoothness, higher rigidity, and increased surface area) improve bone-related biogenesis, propagation, adherence, and accumulation of calcium minerals (Sobieraj and Rimnac, 2009; Gautam et al., 2022). The promising prospective of fabricating orthopedic implants in the future is attributed to the ability of nanomaterials to imitate or reproduce the structural component of natural bone (Cheng et al., 2018b). Bone replacements are employed in orthopedics to address severe damage to natural bones that cannot be restored through the restorative process alone. Nanomaterials may play a major role in this area by providing structural support for cells (via, for example, nano-functionalized scaffolds) and thereby affecting cell differentiation, migration, and proliferation (Patel et al., 2016; Vieira et al., 2017). Characteristics of an ideal scaffold include the ability to support the desired tissue structure, mechanical strength, cytocompatibility, regulated biodegradability, and high biocompatibility (Suh and Matthew, 2000). Aside from that, an optimal scaffold should possess certain specific structural and chemical characteristics. Firstly, it should exhibit an architectural design tailored to meet the requirements of shape, volume, and mechanical strength in three dimensions (Park et al., 2006). Secondly, the scaffold should be highly porous along with interconnected open pores, facilitating the infiltration of cells and the integration of new tissue. This feature promotes a dense cell population within the scaffold and supports effective tissue ingrowth. Thirdly, its chemical composition should minimize immune or inflammatory responses by ensuring the biocompatibility of surface and degradation products (Chung and Park, 2007). Lastly, the scaffold’s degradation rate should be precisely regulated to provide adequate support to impaired tissues until they undergo complete regrowth. To facilitate simultaneous tissue regeneration and replacement, the degradation performance of the scaffold must align with the regenerative rate of the affected tissue, given that the scaffold serves as a temporary matrix for cell differentiation and proliferation (Chung and Park, 2007). Hence, Nanosized structures such as metal-organic frameworks, nanoflowers, nanorods, quantum dots, nanocubes, nanotubes, and nanopillars that are used in implants are very important to consider. Various studies have investigated the advantageous surface characteristics of nanosized ingredients that can stimulate or enable a substantial proportion of precise protein interactions, enhanced osteoblast rise, and enhanced osteoblast progression and movement for effective bone development in comparison to conventional tools (Tran et al., 2009; Marew and Birhanu, 2021). This article discusses the development of various types of nanomaterials used in orthopedic applications. Orthopedic therapies are highly dependent on the accurate localization of therapeutic sites and efficient implantation. To offer a comprehensive overview of the rapidly evolving scientific field, recent advancements in core orthopedic biomaterials, including porous materials, nanocomposite materials, and smart biomaterials, are discussed.

## 3 Current developments of nanotechnology in orthopedic implants

The development of nanotechnology has led to the fabrication of a multitude of nanophase (100 nm particle size) elements, which include metals, ceramics, polymers, and composites. Several of these materials exhibit improved osseo-integration and the ability to develop new bones (Zhang and Webster, 2009; Lowe et al., 2019). It has been reported that the reduction in titanium particle size from 4,500 to 200 nm (produced by analogous channel angular pressing) resulted in a 20-fold increase in cell proliferation. (Lowe et al., 2020). A high density of grain boundaries is a characteristic property of nanophase components due to their different atomic structures. Nanocrystalline materials, which are polycrystalline solids with extremely small crystallites of just a few nanometers in diameter, provide both high hardness and exceptional strength. However, they are brittle and/or ductile (Koch, 2003; Li J. et al., 2020). It is crucial to consider that the lack of elasticity in nanoscale materials may provide extremely challenging issues in the context of advanced structural applications. Numerous factors contribute to the brittleness of nanostructured materials, including their compact production and simple structure (Yang H. et al., 2019). The orthopedic implants demonstrated typical nanostructures (Webster and Ejiofor, 2004; Zhou and Lee, 2011; Costa et al., 2012). Zhang et al. found improved mechanical properties in MgAl_2_O_4_ ceramics-based nanomaterial (40 nm grain size) produced through high temperatures and pressure sintering. These nanomaterials exhibited a hardness value of 31.7 GPa and a young modulus equivalent to 314 GPa (Zhang and Webster, 2009). The nanostructured Ti_6_Al_4_V metal manifested improved mechanical attributes in comparison to pure titanium (Serra et al., 2013). The surface texture virtues for standard titanium and three nanoscale components (Ti_6_Al_4_V, Ti, and CoCrMo alloy) were respectively demonstrated as 4.9, 11.9, 15.2, and 356.7 nm. The roughness of nanostructured materials had a substantial impact on osteoblast function. Research has demonstrated that utilizing different nanoscale materials, such as Ti_6_Al_4_V, Ti, and CoCrMo can enhance osteoblast functions while simultaneously decreasing competitive cell functions (Liu et al., 2014). Another composite used n-HA with polyamide, producing n-HA crystals having diameters of 5–26.7 nm and 30–84 nm long. The content of n-HA reached 60% in the composition, almost similar to natural bone. Stress shielding caused by a mismatch in mechanical properties between the graft and bone can be reduced or eliminated when using n-HA/PA because its Young’s modulus is similar to that of natural bone (Tasker et al., 2007). Additionally, it has been suggested that the integration of CNTs into matrices composed of polycarbonate-urethane (PCU), polycaprolactone (PCL), or polystyrene (PS) could improve the mechanical characteristics of the composite scaffolds, specifically their tensile and compressive moduli (Lalwani et al., 2013). A decrease in degradation rate and an increase in tensile strength of approximately 12% were observed in one study where single-walled CNTs (SWCNTs) were incorporated into poly-L-lactic acid (PLLA) composites. Additionally, a decrease of approximately 5% in polymer crystallinity was observed (Mackle et al., 2011). It was reported that CNFs/polycarbonate urethane composites possessed remarkable mechanical properties, including an elastic modulus of 22 MPa, a tensile strength of 9 MPa, and an elongation of 452% (for 90:10 wt%) (Webster et al., 2004). The enhanced chemical, physical, and mechanical characteristics of nanocomposites are often ascribed to the additive contributions of individual components, in contrast to the properties observed in other materials. Specific applications can be achieved by optimizing the properties of nanocomposites due to their versatile nature. For instance, the distinct configurations of PLA-HA nanocomposites exhibited variations in tensile strength: for example, 840 ± 330 N/mm^2^ for PLA-HA (20 wt%), 770 ± 350 N/mm^2^ for PLA, and 1,030 ± 390 N/mm^2^ for PLA-HA (50 wt%). Similarly, a distinct differentiation was observed in the porosity values 80% ± 3%, 91% ± 2%, and 70% ± 4%, for these three forms (Morelli et al., 2015). To exploit the biodegradable characteristics of the polymer and the exceptional mechanical characteristics of the CNFs, Elangomannan et al. (Elangomannan et al., 2017) constructed CNF/PCL/mineralized HA nanofibrous scaffolds onto a Ti substrate. This resulted in the scaffold exhibiting a favorable elastic modulus and adhesion strength.

### 3.1 Metallic and metallic oxide nanoparticles

Tissue and implant engineering have also benefited from the utilization of nanotechnology. The ability to modify the material’s surface texture and increase its surface area at the nanoscale is expected to result in improved osteogenic cell responses and efficient mechanical contact between the implant and tissue. There are three potential applications for metallic and metallic oxide nanoparticles in the field of bone science: delivery of bioactive molecules, labeling of cells, and improvement of orthopedic implants and scaffolds. The size (typically between 10 and 100 nm) and substantial surface area of nanoparticles render them optimal for the transportation of bioactive molecules, including genetic materials, pharmaceuticals, and growth factors (Kyllönen et al., 2015). Furthermore, orthopedic scaffolds and implants are capable of releasing metallic and metallic oxide nanoparticles that exhibit rapid responses to environmental stimuli, including but not limited to temperature, pH, magnetic field, and other relevant parameters (Walmsley et al., 2015). The integration of nanoparticles with orthopedic scaffolds or implants has the potential to enhance the rate of bone regeneration and facilitate targeted treatment. In conclusion, it is imperative that orthopedic scaffolds and implants possess adequate mechanical strength and biocompatibility. The exceptional mechanical and biological properties, as well as their potent antimicrobial capability, have contributed to the widespread application of nanoparticles in bone-related research (Grass et al., 2011). Specifically, because numerous commercial implants lack antimicrobial potential, which may result in infection or implant failure (Coelho et al., 2019a). To inhibit the development of bacteria, orthopedic scaffolds, and implants may be loaded with drugs like antibiotics. Therefore, it is possible to provide orthopedic implants or scaffolds with sustained antimicrobial activity by coating them with metallic and metallic oxide nanoparticles. In addition, metallic oxide and metallic nanoparticles can be utilized to strengthen scaffolds or enhance the growth rate of bone-related cells at specific dosages (Khoshroo et al., 2017; Zhu et al., 2019a). Consequently, these nanoparticles possess the capability to perform numerous functions concurrently. Orthopedic implants for internal fixation and weight-bearing are typically comprised of metals and alloys. To ensure load-bearing capabilities at the implantation location as well as minimal movement between the implant and host tissue, these implants are firmly attached to bones. Despite the extensive availability of these materials, only a small number of them possess the required biocompatibility and are therefore being successfully employed in long-term implant applications. For example, surgical-grade stainless steel is employed in temporary implants (like fracture plates and hip nails), titanium is used in joint and bone replacement, magnesium is used for load-bearing in biodegradable orthopedic implants, and cobalt-based alloys are used in orthopedic prostheses (for shoulder, hip, and knee) (Bandyopadhyay et al., 2019; Julmi et al., 2019; Zhang and Chen, 2019; Zlotnik et al., 2019). Specific mechanical characteristics are necessary for (i) stabilizing or promoting the fracture integrity, (ii) replacement of joints, and (iii) realignment of bone fragments in orthopedic usage of biomaterials. Patients with diverse medical conditions necessitate orthopedic scaffolds and implants to fulfill their specific mechanical and biological demands. During bone reconstruction, several clinical challenges are faced by surgeons due to patients’ varied anatomical sites, defect sizes, physical conditions, mechanical characteristics, and other factors. (Polo-Corrales et al., 2014). Research on bones has benefited from recent advancements in biomaterial sciences, bio-manufacturing, nanotechnology, and tissue engineering (Mohammadi et al., 2018). The characteristics of metallic and metallic oxide nanoparticles, including gold, silver, magnesium, and zirconia, among others, have been thoroughly documented by numerous researchers.

#### 3.1.1 Magnesium and magnesium oxide nanoparticles

Magnesium (Mg) is an essential component required by the human body. Mg deficiency can inhibit bone growth, cause osteopenia and bone fragility, and reduce bone-related cell activities (Belluci et al., 2011). Mg is distinguished from other metals by several unique characteristics, including its ability to promote bone formation, biocompatibility, biodegradability, and a low Young’s modulus (Meenashisundaram et al., 2020). Mg’s tensile characteristics resemble closely to those of human cortical bone and its elastic modulus and density are very comparable to the bone, rendering it an outstanding material for use in medical implants (Brar et al., 2009). Mg-based implants degrade internally due to the biodegradable nature of Mg, thereby eliminating the necessity for further operations (Witte, 2010). It also decreases the toxicity toward the human body and stops the stress-shielding effect, which refers to the decrease in bone density (known as osteopenia) caused by the absence of normal stress on the bone due to an implant. This phenomenon occurs due to a disparity in material properties between the bone and the implant, resulting in shear stresses (Kirkland, 2012). Through an *in-vivo* investigation on rats, Kraus et al. examined the effect of Mg-based implants on growth plates (Kraus et al., 2018). In the right femoral bone of rodents, close to the growth plate, a Mg implant was inserted. Then holes were made in their bodies for the opposing femur bones. Zn (5%), 0.25% Ca, and 0.15% Mn were alloyed into a Mg implant. According to the results, the alloy maintained its yield and mechanical strengths and degraded uniformly while emitting a negligible amount of gas. Consequently, it satisfies the requirements for application in orthopedics, where fixation nearby of the growth plate is essential.

**FIGURE 1:**
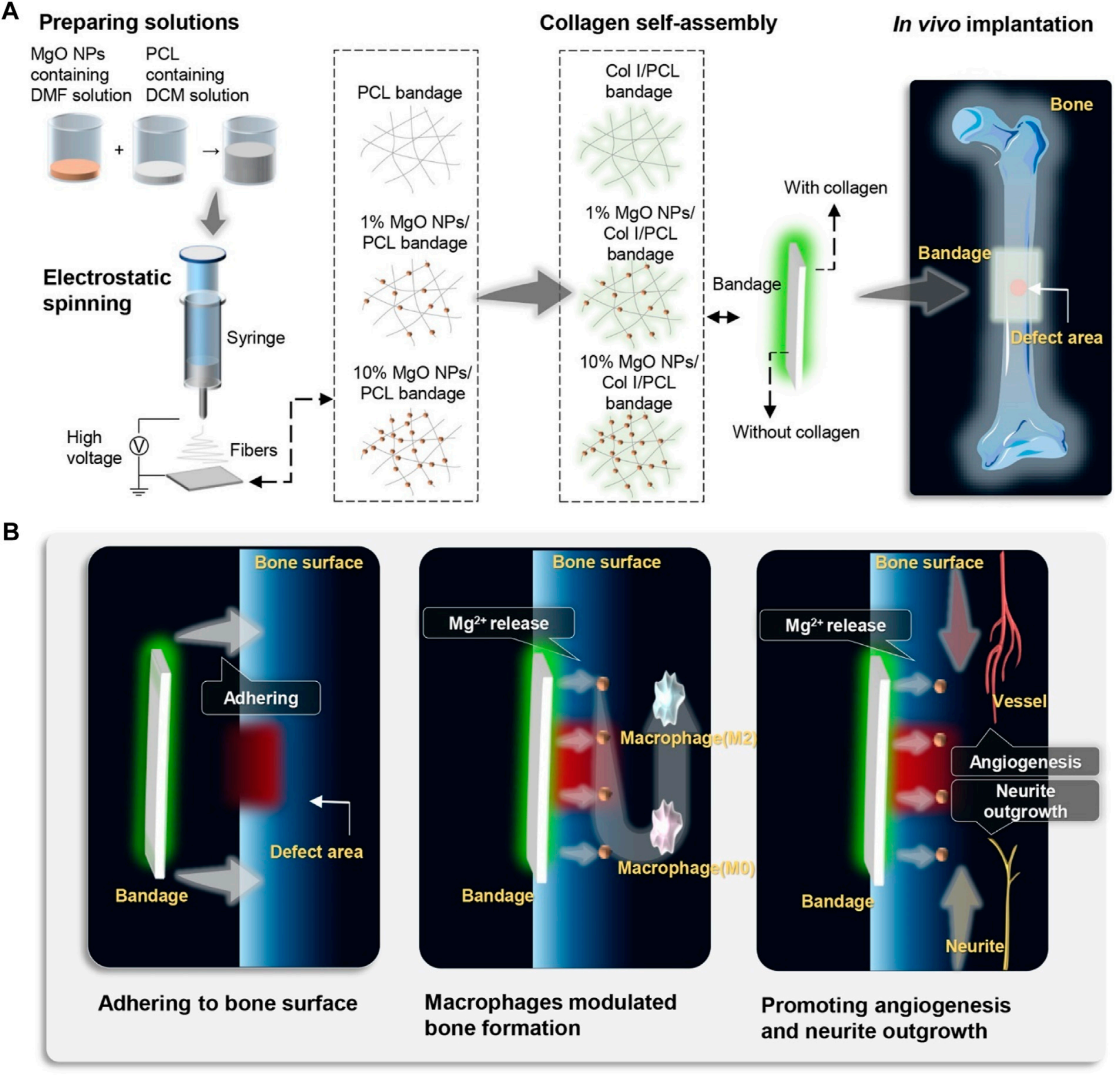
Schematic illustration of the fabrication, *in vivo* application, and mechanism of MgO NPs-carried artificial periosteal bandage. **(A)** An artificial periosteal bandage containing MgO NPs was developed via electrospinning. Prior to *in vivo* implantation, PCL collagen and MgO NPs were self-assembled onto the surface of the periosteal bandage in situ. **(B)** Following *in vivo* implantation, the periosteum bandage remained adhered to the surface of the femoral bone in mice, releasing Mg^2+^. The release of Mg^2+^ directly activated M2 macrophage polarization, promoting angiogenesis and neurite outgrowth, thereby accelerating bone defect healing.

Due to their antimicrobial properties, cheap cost, and environmentally friendly nature, magnesium oxide (MgO) nanoparticles are very useful. Due to their antimicrobial properties, MgO nanoparticles have been used in bone replacements alongside hydroxyapatite to avoid dental and orthopedic infections (Coelho et al., 2019b). Mg nanoparticles have been used in a variety of ways by researchers to address the drawbacks of current scaffolds. To increase its mechanical strength and antimicrobial capacity, Nasir-Nasrabadi et al. fabricated a porous scaffold from sodium alginate along with the incorporation of MgO nanoparticles. (Nasri-Nasrabadi et al., 2018). Mg nanoparticles were added by Dittler et al. to a scaffold based on the bioactive glass to improve its antibacterial properties and biological activity (Dittler et al., 2019). A magnesium-based prosthetic bandage was prepared by Li et al. to stimulate periosteum-associated biomineralization. (Li et al., 2022). A Mg-based artificial bandage was used in a cortical bone defect model to direct newly generated bone tissue to cover the deformed area. They concluded that the suggested periosteal bandages serve as a bioactive medium assisting accelerated bone healing and bone formation can be strategically stimulated via *in situ* Mg delivery to the periosteum (Figure 1).

Mg nanoparticles have received considerable recognition for their low toxicity. Mg nanoparticles, nevertheless, can damage DNA, either directly or by bringing about inflammatory and ROS reactions in cells that lead to cell death or necrosis (Mahmoud et al., 2016). MgO nanoparticles, discovered by Ghobadian et al. possessed the capability to cause cellular death, DNA damage, and intracellular ROS production at various concentrations (Ghobadian et al., 2015). Osteoblastic SAOS2 cells can also be damaged by Mg nanoparticles in a dose- and time-dependent way (Kim and Gilbert, 2019). However, there has not been much study on the toxicity of Mg and MgO nanoparticles, and more research is required to fully comprehend the exact mechanism leading to Mg toxicity.

#### 3.1.2 Tantalum and tantalum oxide nanoparticles

One of the most biologically inert and chemically stable biometals used to fabricate medical implants is tantalum (Ta). It exhibits good biocompatibility and anti-corrosion properties (Mohandas et al., 2014). Unlike any other implantable substance, it had all the benefits of a conventional bone graft, making it highly advantageous for orthopedic implants. Subsequently, the utilization of Ta increased, including its use in mesh for hernia surgery, tubes for the reconstruction of the frontal sinus, and foil for the reconstruction of peripheral nerves (Cristea et al., 2015). Porous Ta can be used to simulate osseous tissue because it has a trabecular shape and open cellular structure. Ta has interconnected holes throughout its structure, which gives it an elasticity modulus resembling cancellous bone (Li et al., 2020). In comparison to conventional metals, porous Ta also offers a considerably higher fixation strength. Shi et al. reported a pedicle screw with a Ta coating and investigated its characteristics (Shi et al., 2017). The results revealed that Ta had great biocompatibility and good mechanical properties. The use of Ta-based screws in therapeutic settings was determined to be possible. Balla et al. developed frameworks composed of porous Ta and investigated their mechanical properties (Balla et al., 2010). The results indicate that the tensile properties of porous Ta resemble those of human bone. Ta samples outperformed Ti samples in the MTT assay for living cell density and demonstrated good cell adhesion.

Tantalum oxide (Ta_2_O_5_) nanoparticles show remarkable radiopacity, chemical inertness, and biocompatibility, rendering them highly suitable for application as contrast agents in CT and X-rays (Bonitatibus et al., 2010; Miao et al., 2019). Tantalum oxide nanoparticles were successfully used by Freedman et al. as a CT contrast agent for visualizing articular cartilage in humans (Freedman et al., 2014). Due to the Coulombic pull, the cationically charged tantalum oxide nanoparticles showed a greater affinity towards articular cartilage. Moreover, Ta_2_O_5_ nanoparticles can enhance the mechanical properties of the bone substitutes. Researchers used Ta_2_O_5_ nanoparticles to strengthen PEEK scaffolds, leading to significant enhancements in Young’s elasticity and compressive strength (Lu et al., 2015). After being exposed to Ta_2_O_5_ nanoparticles, Ta-PEEK showed improved osteogenic differentiation of rBMSCs, *in vitro* and *in vivo,* in addition to mechanical characteristics that resembled closely to those of human cortical bone. Therefore, the incorporation of Ta-reinforced PEEK material rendered it more suitable for orthopedic implant applications compared to standard PEEK material (Zhu et al., 2019b). Ta nanoparticles were used in experiments by Kang et al. with MC3T3-E1 mouse osteoblasts; the Ta nanoparticles revealed high biocompatibility and osteogenic properties and facilitated the proliferation of the MC3T3-E1 osteoblasts (Kang et al., 2017). Furthermore, according to other studies, Ta nanoparticles can cause osteoblasts to engage in autophagy, which enhances osteoblast growth and differentiation while shielding them from ROS damage and apoptosis (Yang et al., 2014; Camuzard et al., 2016). However, the mechanical properties of Ta, including its considerably high elastic modulus (186 GPa) and density (16.6 g/cm^3^), render it incompatible with bone tissue and unsuitable for load-bearing implants (Ore et al., 2021). Additional drawbacks of using Ta as medical implants include their high production cost, donor-site morbidity, poor stability, inability to integrate with the bone, and potential for the transmission of infectious diseases to our bodies (Kim et al., 2013; Putrantyo et al., 2021).

#### 3.1.3 Titanium and titanium oxide nanoparticles

Due to their excellent mechanical and physical properties, commercially pure titanium (Ti) and its alloys are now frequently used in the development of orthopedic and dental implants (Mishnaevsky et al., 2014). Ti-based materials are suitable for bone replacement due to their excellent tensile strength, improved corrosion resistance, high specific strength, rigidity, fracture toughness, and biocompatibility (Sidambe, 2014). Due to its poor electrical conductivity, which results in the formation of a thin passive oxide layer, it is regarded as biocompatible. This oxide coating serves as a protective barrier, effectively mitigating corrosion on the implant surface. Olmedo et al. investigated the localized corrosion effects on Ti implants in rodents (Olmedo et al., 2008). To get proof of pitting corrosion, they stored the implant for 1 minute in a NaCl electrolyte cell first. These were then cleaned before being inserted into rodents’ bodies. The findings showed that the corrosion-induced artificial pitting decreased the apposition of bone. Corrosion had negative impacts because of the release of toxic metal ions into the rats’ bodies. It was determined that corrosion causes Ti to have a bad effect on the body, so pure Ti should not be used to prepare long-lasting medicinal implants.

**FIGURE 2:**
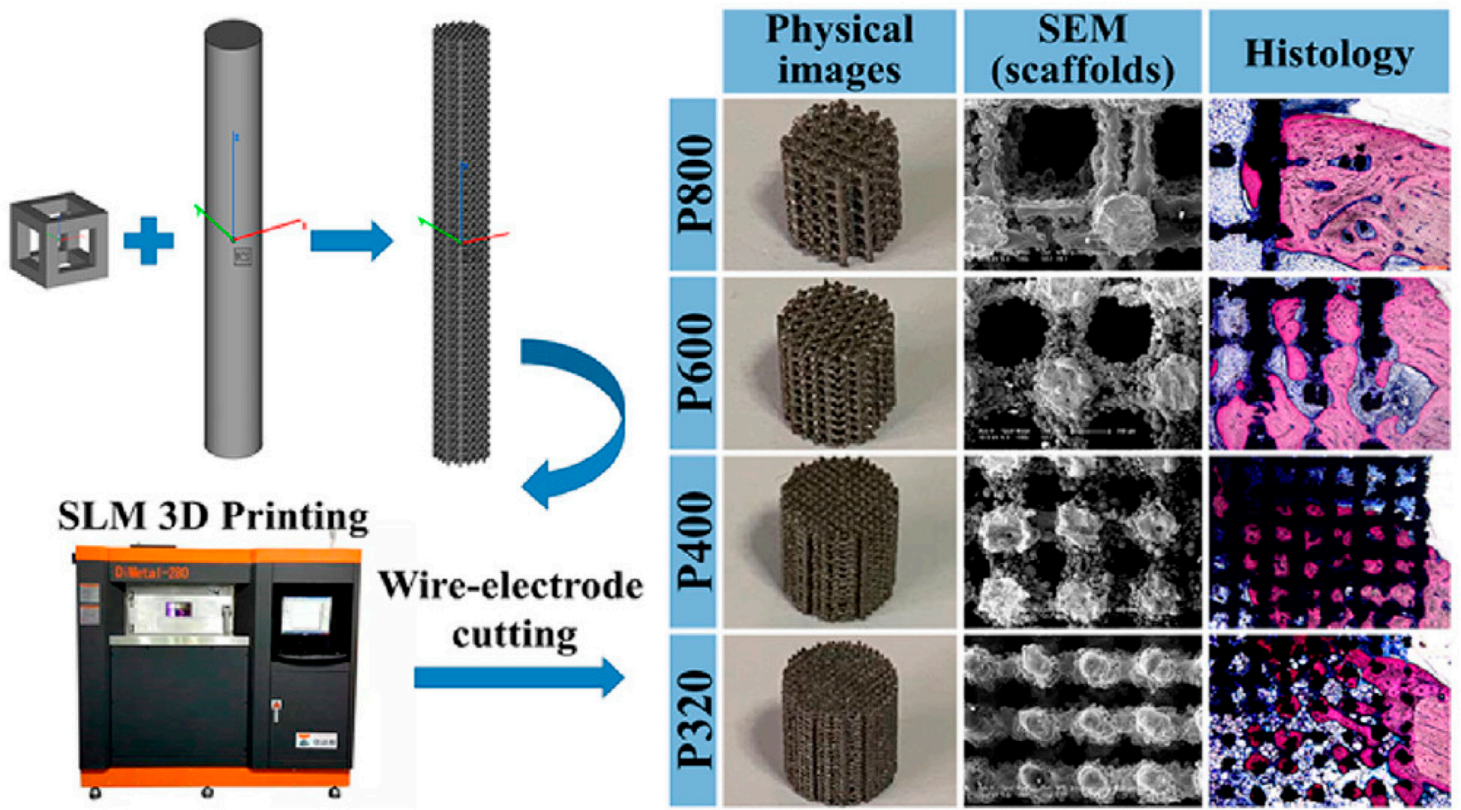
Schematic representation for the preparation of four scaffolds with different pore sizes. Physical images showing the stereo view of scaffolds after being cut into 6 mm high cylinders. SEM images showing the top view of scaffolds. Histological visualization of the bone growth within the scaffold. Reproduced with permission from ACS 2020 (Zheng et al., 2020).

Titania nanoparticles have antibacterial properties against bacteria and fungi, similar to various other metallic nanoparticles (Anwar et al., 2018). They can cause bacterial cell membrane integrity to be compromised, produce ROS on the bacterial surface, and ultimately lead to the release of bacterial components (Priyadarshini et al., 2020). Under certain circumstances, titanium or titania nanoparticles can be beneficial. To modify the surface chemistry, topography, and wettability of a hybrid material and thereby improve osteogenic differentiation and other properties, polymeric matrices have been incorporated with titania nanoparticles such as silk fibroin and titania nanoparticles were combined by Kim et al. to develop scaffolds with an extremely porous structure (Kim et al., 2014). Similar investigations have revealed that incorporating titania nanoparticles can enhance the wettability and compressive stiffness of scaffold surfaces, thereby improving cell adhesion (Rasoulianboroujeni et al., 2019). Zheng et al. (Zheng et al., 2020) effectively fabricated four titanium alloy scaffolds using the selective laser melting (SLM) technique, resulting in structures that are compatible with each other and possess a porosity above 70% (Figure 2). The prepared scaffolds revealed actual average pore sizes of 542, 366, 202, and 134 μm, which were named P800, P600, P400, and P320 respectively. The Ti alloy scaffolds with a diameter of 202 μm, exhibited the most robust osseointegration capacity, as confirmed by *in vitro* characterization, *in vivo* experiments, and scaffold mechanics analyses. Although a slight toxic impact of titania nanoparticles was observed beyond a certain threshold limit, the mere existence of titania nanoparticles in scaffolds did not produce any toxic influence on the viability of osteoblasts. To prepare a sponge for bone healing, Ikono et al. combined titania nanoparticles with chitosan (Ikono et al., 2019), and Titania nanoparticles were found to cause a significant increase in sponge tenacity, biomineralization, and bone healing capacity. Fluoridated titania nanoparticles were used in combination with silk fibroin by Johari et al. to make a novel scaffold; the nanoparticles enhanced the mechanical behavior and cell viability (Johari et al., 2018). Moreover, titania nanoparticles may interact with certain proteins to cause hypersensitivity responses around the implant (Mombelli et al., 2018). Depending on their size, the debris or particles may reach the bloodstream or even cells, where they can cause a variety of short-term (inflammatory and allergic responses) and long-term (such as chromosomal aberrations, hypersensitivity, and carcinogenicity) effects (Gobbi and Gobbi, 2018). It can be concluded that due to the widespread use of titanium in orthopedic implants, it is essential to develop viable methods for integrating titanium nanoparticles and alloys into these implants in a manner that ensures both safety and long-term durability.

#### 3.1.4 Silver and silver nanoparticles

**FIGURE 3:**
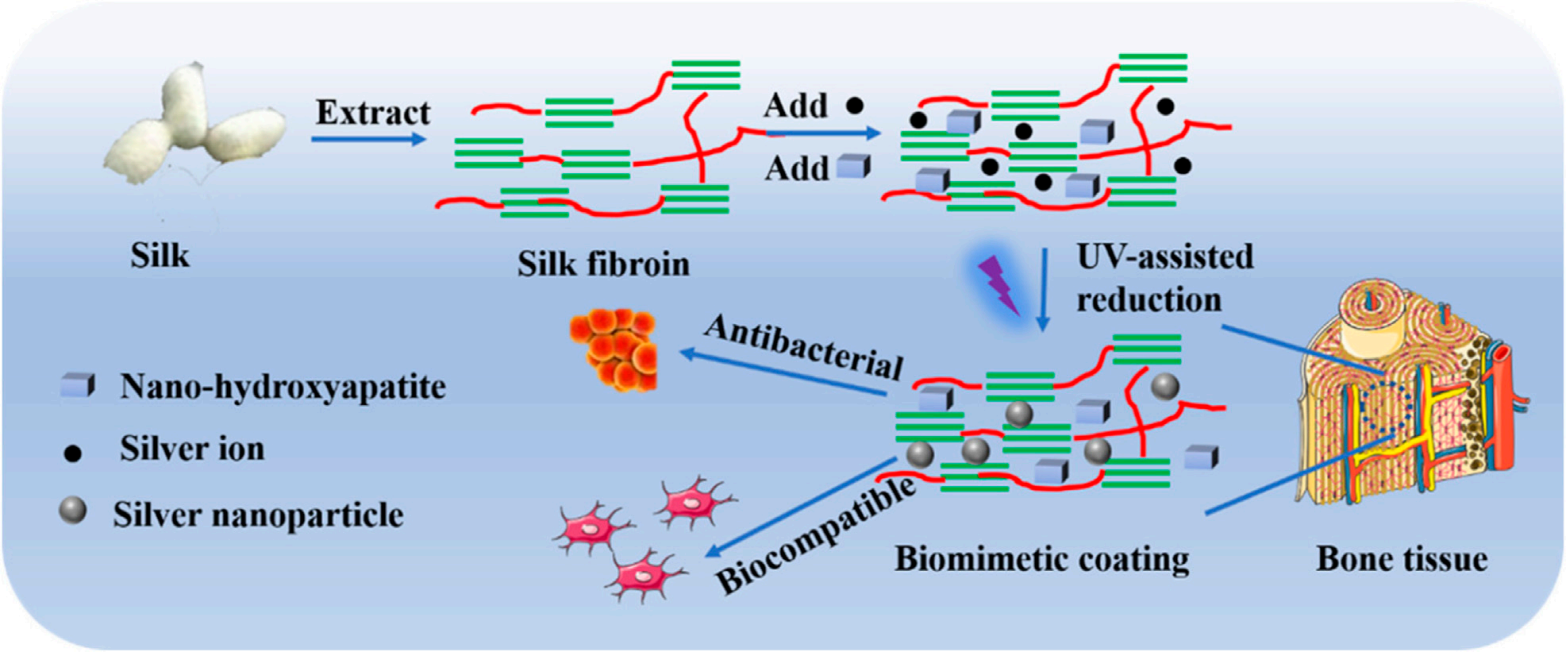
Schematic representation of the procedure involved in developing biocompatible and antibacterial coatings (Zhang et al., 2021).

Silver is favored in bone tissue engineering because of its antibacterial characteristics, but numerous studies have shown that it also inhibits mesenchymal stem cell (MSCs) proliferation (Nguyen et al., 2019). The fundamental analysis to determine a nanomaterial’s biocompatibility is its assessment of proliferation. Although silver nanoparticles can cause cytotoxicity when administered in high doses, it is widely accepted that silver nanoparticles are biocompatible when used in small amounts (Jin et al., 2014). More significantly, silver nanoparticles can provide higher rates of cell division. When silver oxide nanoparticles were combined with titanium oxide nanotubes by Gao et al., the resulting structure had superior cytocompatibility and improved osteoblast growth and differentiation in comparison with the titanium oxide nanotube alone (Gao et al., 2014). According to Zhang et al., silver nanoparticles can promote hMSC growth and osteogenic differentiation both *in vitro* and *in vivo* (Zhang et al., 2015). Silver nanoparticles are capable of penetrating hMSCs and triggering the expression of the HIF-1 gene, which protects the cell against stressful circumstances like hypoxia, ROS production, and other things (Kiani et al., 2013). Silver nanoparticles are also an effective adjunct therapy for bone healing because they can reduce surrounding inflammation. To improve osseointegration and antibacterial activity, Sarraf et al.61 coated tantalum oxide nanotubes with titanium alloy after coating them with a thin layer of silver oxide nanoparticles (Sarraf et al., 2018). In another study, mimicking the natural bone, researchers designed a silk fibroin (collagen-like structure)- coating infused with silver nanoparticles (AgNPs) and nanohydroxyapatite (nHA) to enhance osteogenesis and antibacterial activity. The study placed particular emphasis on the bone mimetic structure as a means to improve bone health (Figure 3) (Zhang et al., 2021). The uniform distribution of AgNPs and nHA on the silk fibroin-based coating prevented *Staphylococcus aureus* from adhering to the surface while simultaneously causing rapid death of planktonic bacteria, demonstrating their potent antibacterial properties. Experimental results conducted *in vitro* demonstrated that the biomimetic silk fibroin-based coating revealed advantageous effects on osteoblast adhesion, spreading, and proliferation (MC3T3-E1) in comparison to uncoated implants. Furthermore, Sun et al. fabricated a novel collagen framework containing bone morphogenetic protein-2 and silver nanoparticles. (BMP-2) (Sun et al., 2015). The antimicrobial ability was increased with the formation of a hybrid structure of the collagen, BMP-2, and silver nanoparticles, and the cell adhesion and proliferation were increased by the surface roughness of the scaffold. A 48 h exposure of Wharton’s jelly-derived hMSCs to ∼30 μg/mL of AgNPs (5 nm) conjugated with fibronectin (FN-AgNPs) has been recently demonstrated to induce proliferation with the production of ROS in a low amount (Hung et al., 2021). Significantly, FN-AgNPs also facilitated the migration of hMSCs and showed anti-inflammatory properties. Since implants often remain within the body for extended periods, continuous research or long-term exposure can elucidate the exact impact of silver nanoparticles (AgNPs) on MSCs. To comprehend the influence of ionic and nano silver, toxicological studies of AgNPs are therefore required.

#### 3.1.5 Zirconium and zirconia nanoparticles

Outstanding antimicrobial properties, biocompatibility, superb corrosion protection, and improved mechanical properties are all characteristics of zirconium, zirconia, and their alloys (Wu et al., 2022; Yang et al., 2022). Instead of being used as nanoparticles, zirconium, and zirconia are frequently used as alloys or coatings in a variety of bio-applications, including biosensors, dental work, and orthopedic devices. Wang et al. used Zn to dope ZrO_2_/TiO_2_ coatings, and they found that zirconia was incredibly durable (Wang et al., 2017) such that only zinc ions were released and no zirconium ions were released. Zinc-doped ZrO_2_/TiO_2_ coatings produced higher antimicrobial activity than zirconia alone, killing significantly more bacteria than zirconia. Since negatively charged bacterial cell membranes interact with Zn^2+^ and Zr^4+^ ions and become damaged, cytosolic leakage and bacterial death result, it is widely accepted that the surface charges of the implant affect their antimicrobial ability (He et al., 2020). Moreover, it was observed that hMSCs were able to develop and proliferate in a healthy environment on the surface of zirconia film (Liu et al., 2006). Zirconia nanoparticles might improve scaffolds’ cytocompatibility, corrosion resistance, and tensile qualities. Yu et al. significantly increased the tensile strength of HA bone cement by adding zirconia nanoparticles to it (Yu et al., 2015). The inertness of zirconium and zirconia may affect their application. To enhance the mechanical properties and refine the osteoconductivity of bone cement additives, Gillani et al. developed functionalized zirconia nanoparticles (Gillani et al., 2010). To reduce the inertness of the zirconia, Bashir et al. developed organic, additive-based zirconia nanoparticles as bone replacements (Bashir et al., 2018). Due to the low expense of glucose and fructose, they employed them as organic additives.

Zirconia nanoparticles have been observed to exert a mild toxic effect. Ye et al. found that exposure of osteoblast MC3T3-E1 cells to zirconia and titania nanoparticles resulted in apoptosis and morphological alterations (Ye and Shi, 2018). Moreover, the authors concluded that zirconia nanoparticles revealed higher toxicity compared to titania nanoparticles. This was attributed to the observation that zirconia nanoparticles induced significantly stronger oxidative stress effects than titania nanoparticles, a factor recognized as crucial in nanomaterial-induced cytotoxicity. Furthermore, higher doses of both could impact osteogenesis and impair osteoblast differentiation. Zirconia nanoparticles require further research and investigation overall because of their enormous potential for use in bone-related applications.

#### 3.1.6 Metal nanocomposites

Numerous metallic and metallic oxide nanoparticles can be toxic to humans and animals over time. A potentially useful approach to reduce nanoparticle toxicity, while retaining their optimum functionality involves the incorporation of one or even more bioactive materials. For instance, Geng et al. added strontium and silver nanoparticles to hydroxyapatite surfaces (Geng et al., 2016). To reduce the possible toxicity associated with silver nanoparticles while enabling the nanoparticles to provide the orthopedic implants with their antimicrobial properties, a secondary dopant strontium was employed. A similar study reported that the addition of magnesium nanoparticles to silver-hydroxyapatite mitigated the adverse effects of silver, leading to an increase in cellular viability (Gopi et al., 2014). Similar to calcium and silver implants, zinc implants with dual loads showed good osteogenic and antibacterial activity (Chen et al., 2023). Tao et al. fabricated a hydroxyapatite-based coating incorporating 10% zinc, magnesium, and strontium ions. This composite coating enhanced the implant’s osseointegration and positively influenced the microstructure of the bone implant (Tao et al., 2016).

*Staphylococcus aureus* is a common source of the progressive infection known as osteomyelitis, which can occasionally result in limb amputation or even death (Masters et al., 2019). Nanoparticles of copper and silver can be employed for its treatment instead of antibiotics. Although the antimicrobial properties of silver and copper together are superior to those of silver or copper alone, their effectiveness diminishes rapidly as copper oxidizes (Bisht et al., 2022). By adding boron, Qadri et al. developed silver-copper-boron (AgCuB) nanoparticles that would retain their antimicrobial properties for an extended length of time (Qadri et al., 2017). It was discovered that AgCuB nanoparticles (1 mg/kg) administered as a single dose could completely wipe out the bacterial population in the infected bone area and retain the antimicrobial potential over an extended duration as a substitute for antibiotics in the treatment of osteomyelitis.

**FIGURE 4:**
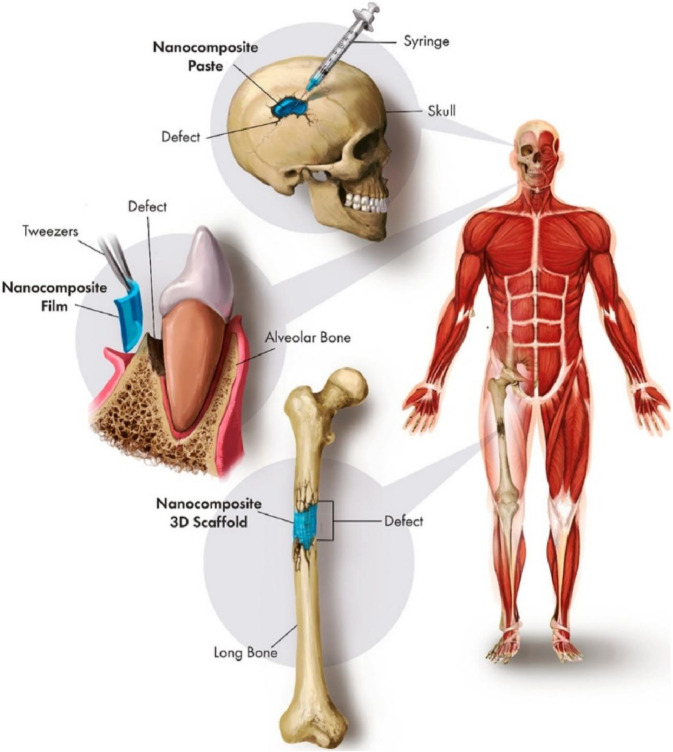
Synergistic use of various nanocomposites for clinical bone defect repair (Lowe et al., 2019).

Other promising research has been conducted to increase bioactivity and corrosion protection, speed up osteoblast proliferation and differentiation, and strengthen antibacterial function through synergistic application of various nanocomposites (Figure 4). A few examples of the combinations are silver and strontium nanoparticles, silver and copper nanoparticles, silver and zinc nanoparticles, and titanium and zinc nanoparticles (Wang N. et al., 2021). To combine and strengthen their beneficial properties, it is rather more sensible to employ numerous nanoparticles in comparison to just one. Safari et al. investigated the potential anti-inflammatory and synergistic effects of graphene nanoparticle modification of Mg alloy (Safari et al., 2020). The addition of graphene nanoparticles into the Mg-based alloys resulted in an about 4-fold decrease in their degradation rate, whereas there was up to a 5-fold increase in the bactericidal activity. Metal, ceramic, and polymeric nanoparticles are often combined as nanocomposites to improve their biological and mechanical characteristics. Compared to monolithic materials and micro-composites, nanocomposite materials, with the ability to mimic the structure of bone while possessing tailored biological and mechanical properties, may represent the optimal choice for fabricating fully functional bone tissues (Shirdar et al., 2019). While considerable research has been conducted on the effects of nanocomposites on bones and their potential applications, further investigation is necessary to fully comprehend their long-term effects both *in vitro* and *in vivo.*

### 3.2 Non-metallic materials

Many non-metallic materials show advanced features suitable for structural implantation. These include crystalline ceramics, polymeric materials, carbon composites, and amorphous glasses. Their poor mechanical properties as well as intrinsic bio-incompatibility, have restricted the widespread use of these materials. Significant efforts have been devoted to enhancing non-metallic materials for their use as structural implants over time (Terzopoulou et al., 2022). The advancement of nanotechnology has facilitated the development of a diverse array of nanophase (100 nm particle size) constituents, including composites, polymers, and ceramics, each possessing distinctive surface properties. Several of these materials exhibit enhanced capabilities in osseo-integration and the formation of new bone (Zhang and Webster, 2009). The more favorable environment provided by nanomaterials for osteoblast function and bone ingrowth results in the formation of a bioactive layer that is adherent, thereby mitigating issues such as implant loosening. To emulate the complex nanostructures found in natural bone, implants’ coatings are modified with nanoscale features, including carbon nanofibers and nanotubes. In joint replacement components, the friction and wear characteristics may be enhanced using nanostructured ceramics. Furthermore, due to their improved osseo-integration and bone regeneration capabilities as well as their ease of fabrication, biocompatibility, flexibility, and appropriate electro-mechanical characteristics, polymer-based materials are favored for (i) controlled drug delivery vehicles. And (ii) porous tissue engineering scaffolds (Al-Shalawi et al., 2023). Polymers, as opposed to metallic implants, possess the capacity to (i) progressively shift stress to a region that has been damaged, enabling appropriate tissue healing, and (ii) restore the function of tissue naturally without the need for catalysis or enzymes (Jain et al., 2022). The material that interfaces the human body and the implant device has to be carefully selected before the installation of implants in the body. These materials should have the capability to impede the transfer of waste material. Polyglycolide (PGA), poly (lactic acid) (PLA), polyhydroxyalkanoates (PHAs), ultra-high molecular weight polyethylene (UHMWPe), polyvinylidine fluoride (PVDF), and polyether ether ketone (PEEK) are the most frequently employed polymers for packaging of orthopedic implants (Gautam et al., 2022). A self-reinforced poly (lactic acid)-b-poly (lactide-co-caprolactone) block copolymer was reported by Wang et al. which manifested enhanced biocompatibility and controlled *in vitro* degradation via the generation of a highly oriented structure for orthopedic application (Wang et al., 2022). Yoon et al. reported a block copolymer consisting of polylactic acid and polyglycolic acid, characterized by excellent biocompatibility and a controlled biodegradation rate. This material was deemed suitable for application in resorbable spinal fixation materials (Yoon and Chung, 2022). Using varying laser powder bed fusion processing parameters, Schappo et al. examined the usage of various concentrations of spray-dried hydroxyapatite particles suspended within a polymeric matrix of ultra-high-molecular-weight polyethylene (UHMWPE) for bone tissue engineering (Schappo et al., 2022). However, the primary issues with polymers involve temperature increase and wear-dependent deformation under loading conditions, which are analogous to corrosive loss observed in metallic implants (Sabir et al., 2009). An appropriate crosslinking technique might be used to tackle UHMWPe’s most prevalent issue, referred to as oxidative deterioration, which is caused by shelf aging. Nevertheless, considering their low metal friction coefficient, they appear to be more suitable for implementation as a bearing surface in total joint devices (Sobieraj and Rimnac, 2009).

Due to their good biocompatibility, superior wear and corrosion resistance, high compressive strength in load-bearing conditions in the human body, and appropriate chemical stability in the physiological environment, ceramics and other non-metallic materials, have drawn significant interest for these applications (Shaaban et al., 2022). Hydroxyapatite (HA), silicon oxide, Calcium phosphates (CaP), and bioglass are the most popular ceramics used in orthopedic devices (Díaz-Cuenca et al., 2022; Montazerian et al., 2022; Van Rijt et al., 2022). CaP ceramics’ strong reactivity and biocompatibility make them particularly appealing as implant coatings. Strong implant fixation and rapid bone development are facilitated by the CaP porous implant coatings. These substances are neither fragile nor easily compressed. The main problem with ceramics is that they have a high noncompliant elastic modulus in contrast to the bone, which may lead to acetabular socket fractures or loosening. These materials’ success relies on their capacity to promote bone growth and regeneration at the tissue-implant interface (Reyes Rojas et al., 2023). Due to their weak ductility and brittleness, they have not yet been utilized for fracture fixation.

Currently, several efforts have been carried out to modify common biomaterials for bone regeneration and fusion with neighboring bone tissues (Thibault et al., 2013). Regarding their ability to precisely direct and impact the functions of cells and tissues in preparation for implantation at a particular location, however, more advancements are still required. Nanotechnology has drawn a lot of focus in the last 10 years due to the development of numerous nanomaterials via various physical, chemical, and biological processes. Natural tissues and organs can readily interact with nanostructured extracellular matrices due to their diverse dimensions (Kumar et al., 2017b; Dogra et al., 2018).

### 3.3 Antimicrobial-coated orthopedic implants

A biofilm is a highly organized, intricate, and spatially organized network where the majority of pathogenic microorganisms that are affiliated with the host can be found (Yazici et al., 2016). The extracellular matrix (ECM) that makes up biofilms comprises the bacteria’s secreted exopolysaccharides (EPSs), extracellular DNA, proteins, and specific quorum-sensing proteins needed for cell-to-cell transmission. Various bacterial populations are packed within the ECM. The surface characteristics of the implant, including hydrophobicity, physicochemical properties, roughness, and charge, play a crucial role in initiating biofilm formation by these microbes. Biofilm formed on implants or medical devices is more resistant to host defense mechanisms as well as antibiotics, in comparison to the free-floating planktonic variety (Saeed et al., 2018). Nanomaterials have become an effective way to eliminate drug-resistant biofilm-associated nosocomial implant infections, medical devices, and other biomaterials to fight the issue of antibiotic resistance in microbes that are involved in biofilm formation. Due to their small size, nanoparticles can easily penetrate the cell membranes of microbes and the EPS layer of biofilms. Moreover, nanoparticles have a high surface-to-volume ratio, which enhances their biological activities and chemical reactivity. This causes irreparable cell damage and, ultimately, cell death (Robino and Scavone, 2020; Sahoo et al., 2022). Nanocoating materials are employed to alter the surface characteristics of intrinsic implants, thereby mitigating the risk of biofilm infections. One strategy is to develop resistance on the surface against the formation of biofilm and to impede the process of protein adsorption to target the first stage of the formation of biofilm, which involves the adherence of planktonic bacteria to the surface of implants. To achieve this, the surface can be coated with antibacterial nanomaterials that inhibit the adhesion of bacteria and possess antifouling or antimicrobial characteristics that eliminate microorganisms coming into contact with the implant or device surface (Shahid et al., 2021).

**FIGURE 5:**
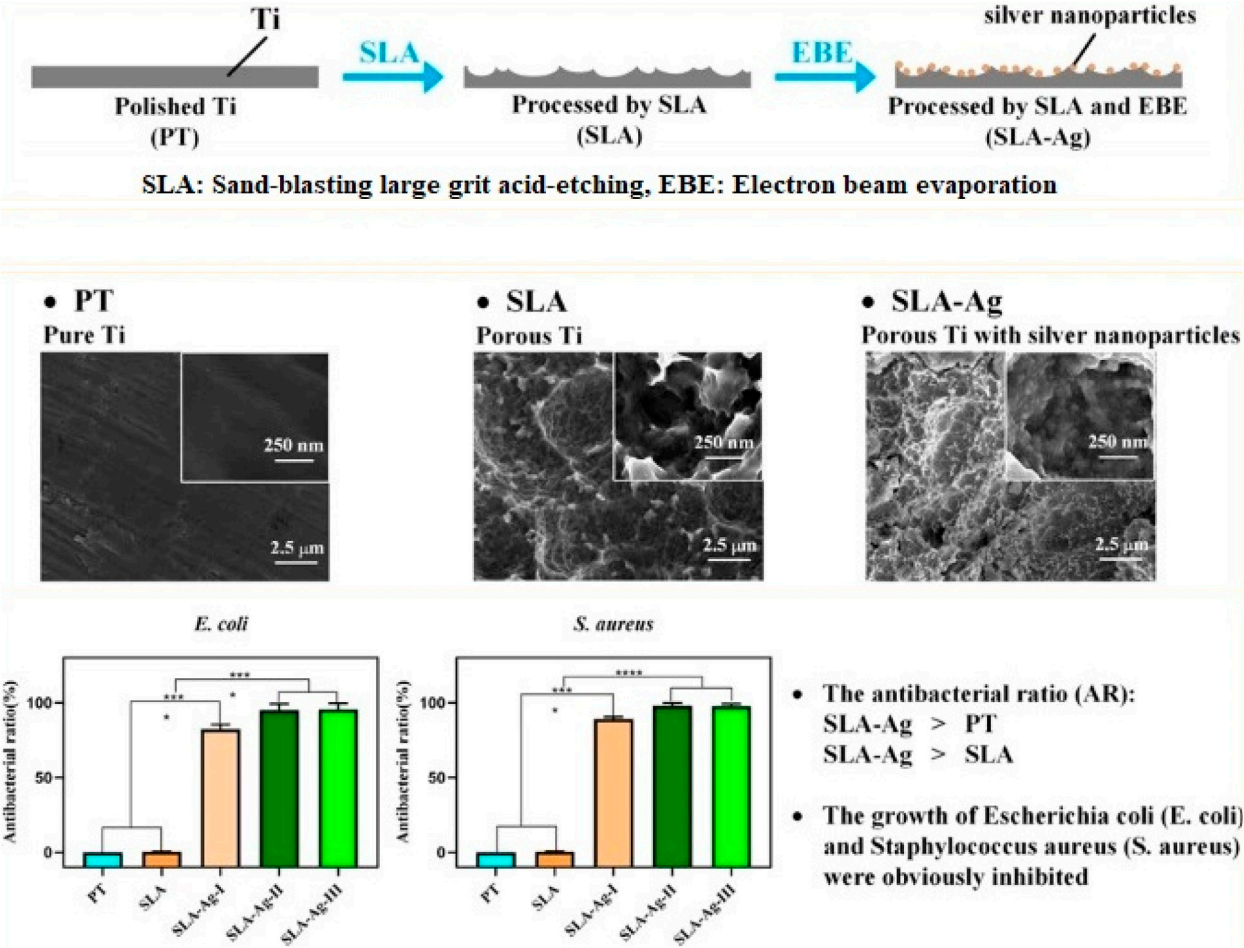
Enhancing antibacterial property of porous titanium surfaces with silver nanoparticle coatings via electron-beam evaporation (Zhang et al., 2022).

Metal nanoparticles are the most widely used inorganic nanoparticles, and they present a promising therapeutic approach against antibiotic resistance. Various types of metal nanoparticles have been shown to enhance antibacterial properties. The most widely used antibacterial nanoagents include gold (Au), silver (Ag), magnesium (Mg), zinc (Zn), titanium (Ti), nickel (Ni), copper (Cu), and their oxide-based nanoparticles (Correa et al., 2020). Due to their high surface-to-volume ratio and smaller dimensions compared to bacteria, metal nanoparticles offer potent, targeted, and prolonged antimicrobial activity along with antibiofilm interactions (Gold et al., 2018; Correa et al., 2020). Recent research by Zhang et al. describes the application of an alkali-heat-treated nano-structured TiO_2_/CuO/Cu_2_O layer to a Ti-Cu alloy that has osteogenic and antibacterial properties. The nanostructured layer facilitated early adhesion, spread of MC3T3-E1 cells, osteogenic differentiation, and showed potent antibacterial activity against *S. aureus.* Zhang et al. suggested a new implant surface modification technique using an easy electron beam evaporation method to coat silver nanoparticles onto the porous Ti surface (Zhang et al., 2022). The growth of *S. aureus (S. aureus) and E. coli (Escherichia coli)* is successfully inhibited by the composite surfaces (Figure 5).

In recent years, the primary concern regarding metal oxide and metallic nanoparticles has been the emergence of microbial resistance to them. Therefore, achieving effectiveness often requires extremely high concentrations of nanoparticles, which, in turn, may pose toxicity risks to animal cells. 2D nanomaterials are used to address the issues with metal oxide as well as metallic nanoparticles. Many diverse 2D nanomaterials are used in antimicrobial coating agents, including black phosphorus, graphene oxide (GO), boron nitride, and MoS_2_ molybdenum disulfide. A catheter with hydrated GO coating fabricated by Ruibin Li et al. and colleagues demonstrated outstanding antimicrobial potential on the density of carbon radicals (Li et al., 2016). In a recent study, it was found that a self-activating implant modified with hydroxyapatite (HA)/MoS_2_ coating can effectively mitigate infections caused by *E. coli* and *S. aureus*. Moreover, this implant stimulates the regeneration of bone tissue by encouraging mesenchymal stem cells to differentiate into osteoblasts via alteration of the mitochondrial membrane and cell membrane potential (Fu et al., 2021). Shaw et al. reported that when applied to medically relevant surfaces, a layer of black phosphorus without solvents exhibited remarkable antimicrobial properties alongside good biocompatibility (Shaw et al., 2021). Ding et al. (2020) reported a titanium-based implant that incorporated silver nanoparticles loaded into mesoporous silica nanoparticles and coated in multilayer layers of polyallylamine hydrochloride and poly l-glutamic acid. This nanomaterial was specifically engineered to combat *S. aureus*-associated infections and to promote *in vivo* bone tissue growth (Ding et al., 2020). They concluded that the reported approach could effectively kill bacteria while promoting osseointegration in an environment infected with bacteria. Recent research by Li et al. has shown that a polyetheretherketone composite modified with black phosphorus and carbon fiber shows superior wear resistance, minimal cytotoxicity, and exceptional antimicrobial properties. These findings suggest that this composite could be an ideal material for implants (Li J. et al., 2022).

### 3.4 Smart orthopedic implants

From nondegradable bone cement to bone graft substitutes crafted from biodegradable materials, bone grafting has emerged as a valuable strategy to address reconstruction needs with improved clinical use. Research on the interactions between cellular material and parameters such as topography, stiffness, porosity, and functional groups provided the basis for the ability to modify cell behavior. Smart/intelligent biomaterials and implants are produced by incorporating these characteristics into biomaterials and their 3D shapes (Intravaia et al., 2022). Smart or intelligent biomaterials are those that have the potential to stimulate tissue regrowth through physical, chemical, electrical, or magnetic stimuli. The goal is to stimulate the production of new bone tissue or osteogenesis, by the progenitor/stem cells either internally or externally using a biomaterial implant (Zhang et al., 2018). These stimuli have been incorporated using cutting-edge biomaterials and/or fabrication techniques. (Zhang et al., 2018).

**FIGURE 6:**
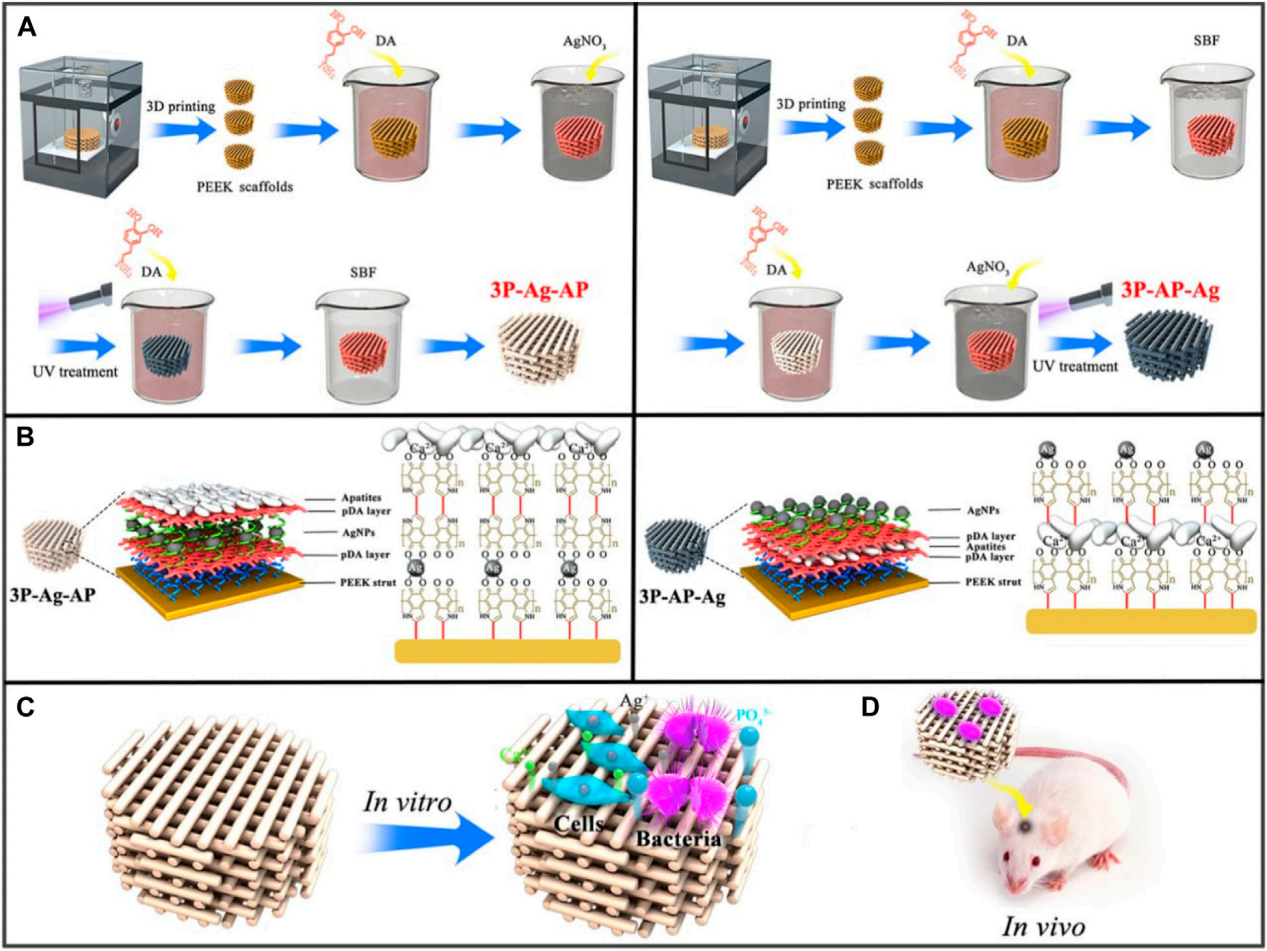
**(A)** Synthesis of 3P-Ag-AP (left), and 3P-AP-Ag (right) scaffolds with pH-triggered osteopotentiating characteristics. **(B)** A schematic depicting the proposed multilayer coating architecture and the potential interactions between the various coating constituents. Schematic of **(C)** *in vitro* and **(D)** *in vivo* tests for the multifunctional scaffolds. Reproduced with permission from ACS 2020 (Deng et al., 2020).

The internal microenvironment contains a variety of biological and chemical features that, when incorporated into the composition of biomaterials, can enable stimuli-responsive bone regeneration (Kim et al., 2021; Wei et al., 2022). Surface chemical interactions, pH, and particular molecules like glucose are examples of chemical stimuli in the microenvironment, whereas enzymes, reactive oxygen species (ROS), and bioactive molecules are classified as biological signals. For example, Deng et al. developed porous PEEK scaffolds having a coating that gave the biomaterial a unique acidic pH-responsive property capable of being triggered by bacteria (Figure 6) (Deng et al., 2020). Recombinant human bone morphogenetic protein 2 (rhBMP-2) and glucose oxidase were grafted onto a new glucose-sensitive controlled-release fiber scaffold fabricated by Jiang et al. to support bone regeneration (Jiang et al., 2022). A titanium bone prosthesis with an immobilized surface that contains both an MMP-9 responsive peptide and the antimicrobial peptide GL13K was developed by Fischer et al. to respond to both MMPs and bacteria (Fischer et al., 2021). In addition to an acidic pH and dysfunctional enzymes, different disease environments exhibit abnormally high amounts of ROS. Higher levels of ROS have been linked to bone-related illnesses like rheumatoid arthritis, osteoporosis, and bone metastases (Ren et al., 2022). In a study conducted by Li et al., a Tb4-loaded titanium implant covered in a multifunctional hydrogel was investigated for its ability to target ROS within a femoral bone defect (Lu et al., 2004). The implant’s hydrogel coating had borate ester bonds in it that can quickly oxidize in the presence of H_2_O_2_, giving it ROS-responsive degradation properties.

**FIGURE 7:**
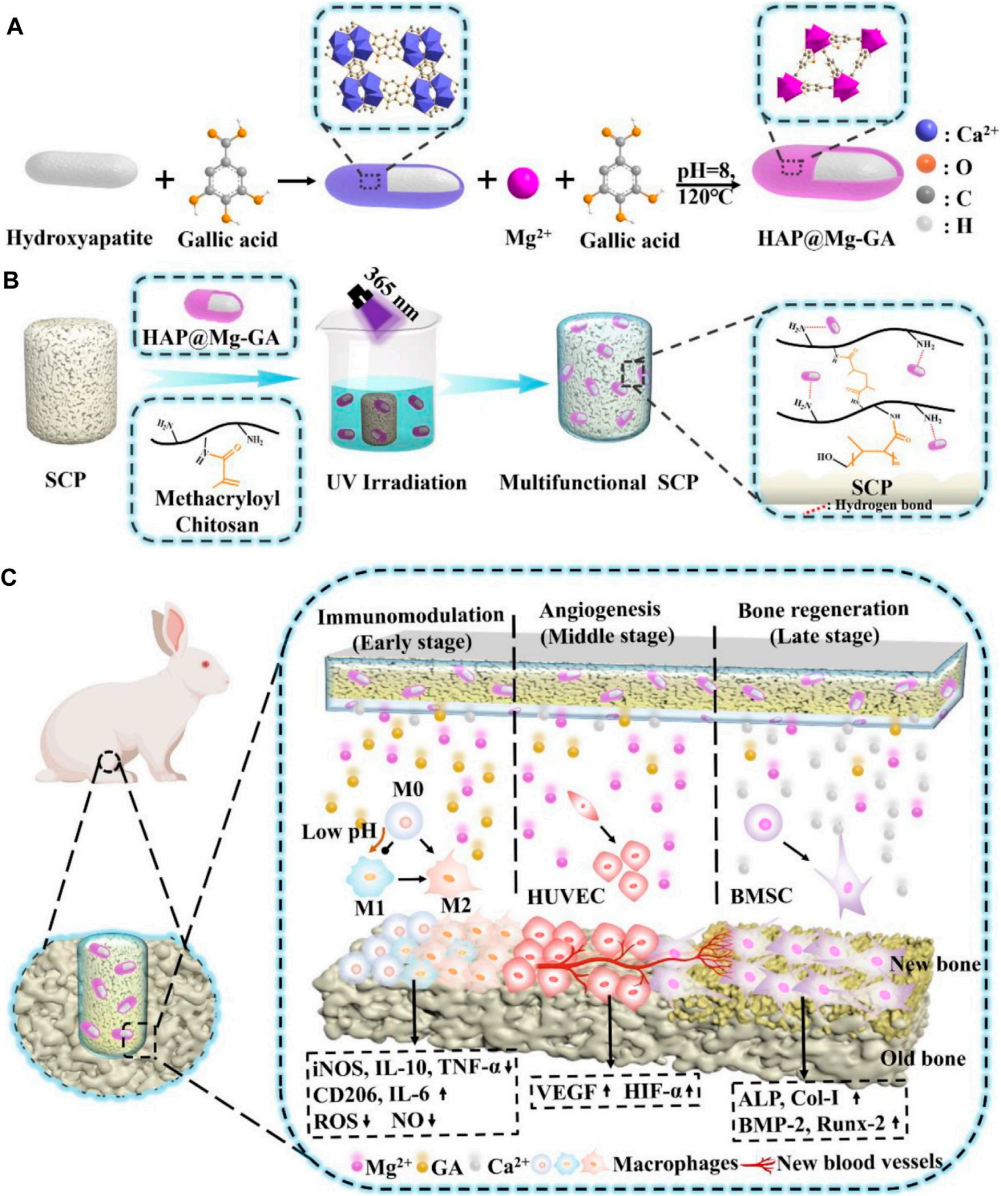
**(A)** Schematic illustration of the synthesis of HAP@Mg-GA MOF. **(B)** Stimulus-responsive sulfonated LCFRPEEK implants comprised of a metal–organic framework hydrogel coating **(C)** Schematic illustration of stimuli-responsive metal–organic framework hydrogel-decorated sulfonated LCFRPEEK implants for enhanced osseointegration (regulation of inflammation response, angiogenesis, and bone regeneration).

While pathological conditions are primarily triggered by internal stimuli, some defect conditions necessitate the targeted administration of therapeutic agents and/or growth factors at a specific dose and time. By adjusting the activity and intensity of the applied stimulus, it is possible to regulate dosage and release periods when using an external source of stimuli. Magnetic fields and light are two frequently used external stimuli for smart biomaterials. Typically, magnetic nanoparticles (MNPs) and photothermal nano-agents can be used to incorporate these stimuli into the architecture of a biomaterial, respectively (Wei et al., 2022). Tanasa et al. incorporated this impact of magnetite nanoparticles into the silk fibroin scaffolds to stimulate and accelerate the osteogenic process in response (Tanasa et al., 2020). It was found that the implanted magnetic nanoparticles affected the distribution of cells around the scaffold and the orientation of actin filaments when a 120 mT magnetic field was applied. Additionally, stimulation by an exterior magnetic field boosted preosteoblasts’ capacity for osteogenesis and their capacity for cellular proliferation. An entirely different approach was adopted by Tang et al. (Julmi et al., 2019) who developed a magnetic responsive bioactive coating that may be added to titanium substrates employed for bone implants (Liu et al., 2011). In this method, CoFe_2_O_4_ nanoparticles were mixed into a P(VDF-TrFE) matrix to form a magnetically active coating. This study provided evidence indicating that nanoparticles of iron oxide do not possess critical properties for magnetically active biomaterials. This finding has paved the way for the exploration of further design concepts that incorporate an iron oxide coating. Being able to regulate the location, wavelength, duration, and intensity of the light source without causing any harm makes light-responsive biomaterials advantageous (Lee and Gaharwar, 2020). Recently, black phosphorus nanosheets (BP NSs), zinc oxide nanowires (NWs), and polydopamine (PDA) were combined with titanium (Ti) substrates to produce a phototherapeutic system. Fang et al. developed a phototherapeutic system showing exceptional antibacterial qualities against bacteria that cause biofilms (Fang et al., 2022). Recently, UV-grafting was used to fabricate multifunctional stimuli-responsive metal–organic framework (MOF) hydrogels on the surface of sulfonated long carbon fiber reinforced polyetheretherketone (LCFRPEEK) implants to facilitate osseointegration (Figure 7) (Dong et al., 2023). Hydrogels consist of methacryloyl chitosan and nano-hydroxyapatite-coated magnesium-gallic acid (HAP@Mg-GA) MOF nanoparticles. The stimuli-responsive MOF hydrogels demonstrate exceptional capabilities for the pH-sensitive release of biomolecules. Moreover, the superior *in vivo* immunomodulatory, angiogenesis, osteogenic differentiation, and osseointegration capabilities of the implant are further supported by the SD rat subcutaneous implantation and rabbit tibia defect models. Therefore, the research advances the effective applications of LCFRPEEK biomaterials in orthopedics by developing a novel multifunctional orthopedic implant capable of osseointegration. To take advantage of GO potential. For osteogenesis and its capabilities as a drug transport system, Wang et al. used NIR light to reduce GO loaded into a hydrogel film based on chitosan and incorporating teriparatide (Wang X. et al., 2021). It has been demonstrated that, in contrast to continuous release, the release of Teriparatide in a pulsatile fashion *in vivo* can stimulate angiogenesis and local bone regeneration in an osteoporotic bone defect (Figure 7). Nevertheless, additional investigation is needed to determine the most efficient pulsatile schedule. A pulsatile delivery schedule demonstrates the potential for automation in the design strategy, which potentially improves the regenerative impact.

### 3.5 Nanotechnology in orthopedics based on drug delivery system

**FIGURE 8:**
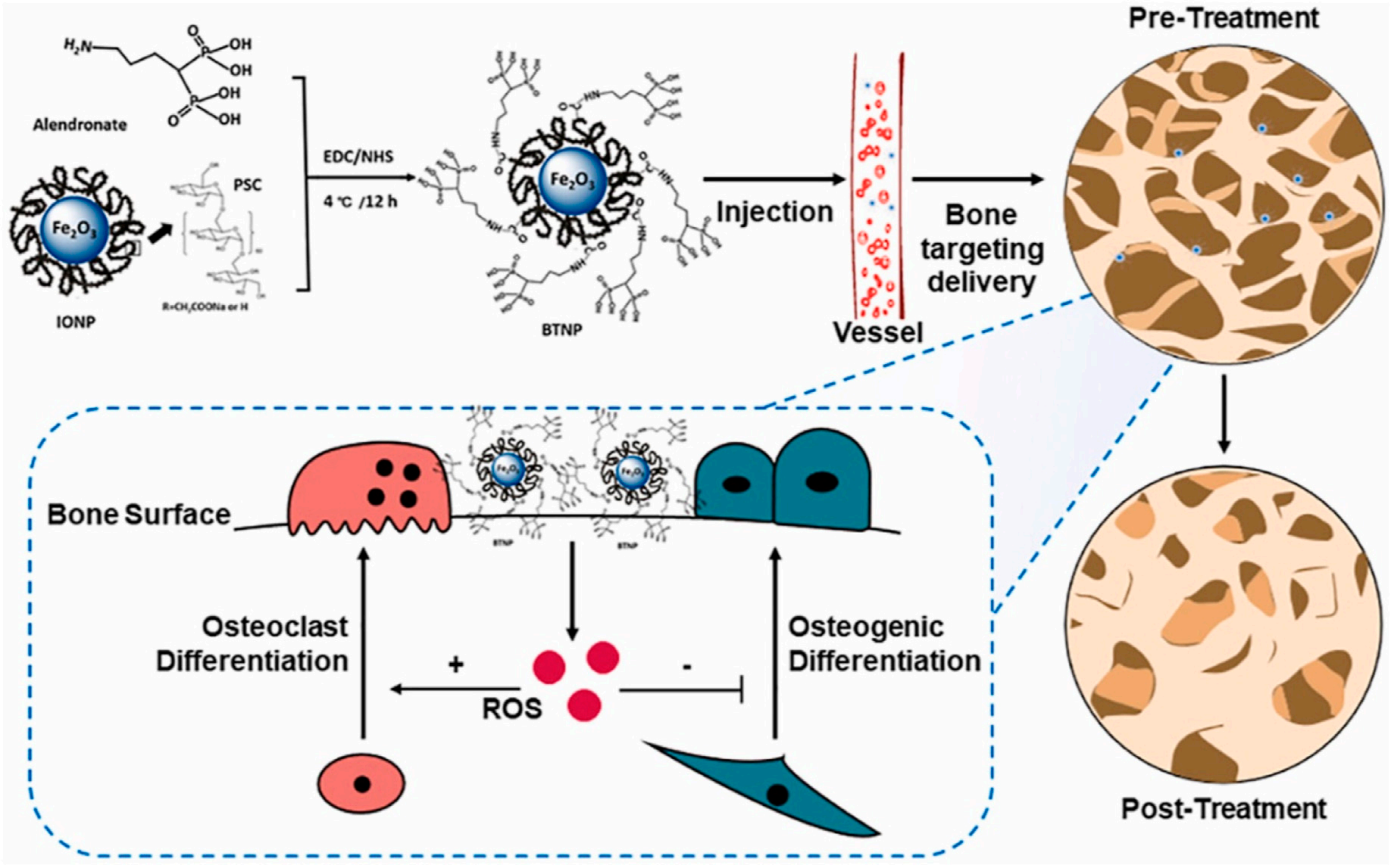
BTNPs were fabricated utilizing alendronate and IONPs. BTNPs administered intravenously to osteoporotic rodents were transported to the bone tissues with precision. By manipulating the local concentration of ROS, the osteogenic and osteoclast development processes were controlled, and OVX-induced osteoporosis was alleviated (Zheng et al., 2022).

In orthopaedics, drug delivery might represent the most significant future application of nanotechnology. Significant progress has been made in the area of nanotechnology-based drug delivery systems (DDS) designed for the specific, prolonged, and targeted delivery of pharmacological substances. These developments have shown improved therapeutic effectiveness and decreased incidence of adverse effects, therefore being especially beneficial in the domain of orthopedics. A wide variety of novel DDS has been developed to encapsulate, transport, and control the release of drugs by employing nanotechnology-derived nanomaterials with unique chemical, physical, and biological characteristics. Conventional pharmaceutical methods are constrained by their inability to overcome particular biological barriers, inadequate solubility, and a lack of specificity, which results in severe adverse effects (Lu et al., 2021). DDS based on nanotechnology have unique characteristics that enable them to avoid the aforementioned limitations. The above-mentioned benefits encompass improved targeting capabilities, which result in decreased toxicity and enhanced bioavailability. Moreover, their nanoscale dimensions strengthen them to overcome biological barriers, whereas their high surface area to volume ratio promotes efficient drug delivery. In addition, interactions with biological targeting molecules are facilitated by the abundance of surface chemistry in these systems (Yang et al., 2020). Innovative advancements have been made in the field of orthopedics through the utilization of nanotechnology-based DDS, including metallic NPs, polymeric NPs, and lipid NPs (Yang X. et al., 2019). Intelligent DDS have been extensively utilized for the identification and treatment of bone-related conditions, including osteoarthritis, orthopedic oncology, osteoporosis, and orthopedic infections, as well as the regeneration of cartilage and bone tissue. The objective of their application is to improve the precision and efficacy of existing therapeutic methods. Millions of individuals suffer from osteoporosis (OP), a prevalent progressive and deteriorating orthopaedic disease. However, adverse effects result from the systemic administration of current anti-osteoporotic medications, including calcitonin, bisphosphonates, and vitamin D. Hence, it is highly desirable and extremely challenging to develop novel DDS that exhibit improved treatment efficacy. Contemporary approaches to treating osteoporosis rely directly on the regulation of bone metabolism. Zheng et al. (2022), utilized alendronate, a clinical bisphosphonate approved by the Food and Drug Administration, to synthesize novel bone-targeting antioxidative nano-iron oxide (BTNPs) (Figure 8). By selectively targeting the surface of the bone, this technology has the potential to positively modulate the *in vivo* equilibrium between bone resorption and formation. Furthermore, employing nanotechnology for drug distribution presents a promising option for OA, a prevalent joint ailment for which therapeutic options are limited. Suboptimal targeting of cartilage after systemic administration and rapid clearance after intra-articular injection are the principal constraints associated with the treatment of osteoarthritis. To overcome these limitations, extensive nanotechnology-based research has been conducted on DDS. The study conducted by Wei et al. demonstrated that the combination of transforming growth factor (TGF) and novel polymeric micellar NPs revealed significant attributes including outstanding biocompatibility, stability, extended joint retention, and efficient cartilage infiltration. Following intra-articular injection, TGF-NPs exhibited a significant level of efficacy in ameliorating cartilage degeneration associated with arthritis, subchondral bone plate sclerosis, and post-surgical joint pain (Wei et al., 2021). Alternative methods of drug distribution have been implemented, including the use of recyclable polylactic acid (PLGA) to administer Kartogenin. PLGA is a small chemical compound that can differentiate mesenchymal stem cells derived from bone marrow into chondrocytes. It has been shown to promote the synthesis of hyaline cartilage and accelerate the correction of defects. (Shi et al., 2016). The potential of nanotechnology-based DDS to enhance the treatment of bone-related disorders has garnered considerable interest. Despite notable progress in the domain of nanotechnology-driven DDS for orthopedics, concerns persist concerning its metabolic pathways and long-term safety.

## 4 Future perspective and concluding remarks

While prelusive research indicates the potential use of nanomaterials in orthopedic applications, additional advancements are required to attain practical applicability. The aim of current research in the realm of bone tissue engineering is to develop biocompatible scaffolds that can function as a partial replacement for normal tissues while also interacting with their surroundings, sensing and reacting to environmental cues, and effectively influencing cellular processes to accelerate bone formation, reduce treatment time, and promote a more rapid return to function. Future efforts are likely to prioritize the advancement of design methodologies that leverage nanomaterials and nanofabrication approaches to enhance performance and functionality. It is essential to have a solid understanding of the molecular mechanisms that lie behind cell-nanomaterial interactions. Moreover, it is essential to exercise caution in assessing the biosafety of nanomaterials and mitigating their potential effects, given valid concerns regarding the toxicity of nanoparticles generated due to wear and strain. Also, the behavior and material properties of metals change significantly from the micro to the nano size, therefore their potential toxicity at the nanoscale must be appropriately investigated.

Therefore, nanotechnology-cured conventional implants with specific features are superior to implants composed of nanoparticles. This takes away the possibility of nanomaterials spreading in the body and causing toxicity. Because of these concerns, several regulations have been suggested as necessary. Companies remain cautious about developing nanostructured implants and prosthetics due to concerns regarding their therapeutic effectiveness, potential toxicity, and the associated high costs. There are concerns about the toxicity of nanoparticles formed as a result of wear and strain. Metals have distinct behavior and material properties at the nanoscale compared to the microscale.

Nanotechnology is still in its early stages of development, but it has the potential to enhance orthopedic diagnosis, treatment, and research. The success of the business and service sectors supports the idea that nanotechnology will play a significant role in future medical treatments. It has the potential to substantially decrease the expenses associated with numerous conventional pharmaceuticals and facilitate the exploration of novel applications that have not been previously considered. Nanotechnology allows for more precise treatment methods, leading to more effective and long-lasting implants, reduced risk of infection, and improved healing of bones and tendons. The potential advantages of nanomedicine, particularly in the realm of orthopedics, have begun to emerge following extensive research efforts. However, additional investigation is required to fully comprehend the safety and utility of this innovative technology.
